# Circle of care modelling: an approach to assist in reasoning about healthcare change using a patient-centric system

**DOI:** 10.1186/s12913-016-1806-7

**Published:** 2016-10-04

**Authors:** Morgan Price

**Affiliations:** 1 Island Medical Program, University of British Columbia, Vancouver, Canada; 2 Department of Family Practice, Medical Science Building, University of Victoria, PO Box 1700 STN CSC, Victoria, BC V8W 2Y2 Canada

**Keywords:** Circle of care, Quality improvement, Modeling, Clinical informatics

## Abstract

**Background:**

Many health system and health Information and Communication Technology (ICT) projects do not achieve their expected benefits. This paper presents an approach to exploring changes in the healthcare system to better understand the expected improvements and other changes by using a patient-centric modelling approach. Circle of care modeling (CCM) was designed to assist stakeholders in considering healthcare system changes using a patient centric approach.

**Methods:**

The CCM approach is described. It includes four steps, based on soft systems methodology: finding out, conceptual modelling, structured discussion, and describing potential improvements. There are four visualizations that are used though this process: patient-persona based rich pictures of care flows (as part of finding out), and three models: provider view, communication view, and information repository view (as part of conceptual modelling).

**Results:**

Three case studies are presented where CCM was applied to different real-world healthcare problems: 1. Seeking improvements in continuity of care for end of life patients. 2. Exploring current practices for medication communication for ambulatory patients prior to an update of a jurisdictional drug information system. 3. Deciding how to improve attachment of patients to primary care. The cases illustrate how CCM helped stakeholders reason from a patient centered approach about gaps and improvements in care such as: data fragmentation (in 1), coordination efforts of medication management (in 2), and deciding to support a community health centre for unattached patients (in 3).

**Discussion:**

The circle of care modelling approach has proved to be a useful tool in assisting stakeholders explore health system change in a patient centric approach. It is one way to instantiate the important principle of being patient centered into practice when considering health system changes.

## Background

Health information communication technology (ICT) can improve quality and efficiency of health care [1]. However, many health ICT systems do not meet expectations. Health ICT can cause unintended consequences or negatively impact decision making [2–4]. Implementation of health ICT has been a costly venture internationally and the risks of implementing are not well predicted or understood [5]. Further, there is a need to make health systems more patient centric [6]. However, despite the need, many tools used to define requirements for health ICT are not patient centric. There are calls to re-examine health ICT policy to better derive benefits by focusing on usability, interoperability and quality [7]. However, to improve the chance of success and to realize benefits, approaches are needed to better engineer health ICTs and complex health systems together to take advantage of the potential benefits of health ICT [8]. Some have suggested that healthcare specific modeling approaches are beneficial [9].

### Objective

The objectives of this paper are to: 1) describe the *circle of care modelling* (CCM) approach that is patient centric and 2) show its use in exploring healthcare issues so that others may consider its use.

First, we describe related modelling approaches. In materials and methods, we describe the CCM approach. In results, we illustrate use of the CCM with three case reports. Finally, the discussion summarizes and highlights other considerations when applying CCM.

### Related work

There are several modelling approaches that have been developed and applied to health ICT. Unified Modelling Language (UML) is a general purpose, object oriented modelling language that is used to model software systems. There are 14 UML diagrams, which have been divided into two broad types: structural and behavioural [10]. SysML is a subset and extension of UML that is designed to better model socio-technical systems than UML [11, 12].

Patient journey mapping is a form of process mapping that is centered on the path that the patient takes through the healthcare system [13]. It uses flowcharts or process maps to illustrate the journey and highlight decision points and is often used to discuss opportunities for process redesign [14, 15]. It has been used in several areas, such as to explore transitions of care from primary to acute care and back [16].

Soft Systems Modeling (SSM) was developed to explore complex socio-technical problems with systems thinking [17, 18]. SSM follows a four step process of (a) finding out about the real-world problem, (b) select conceptual modeling the system (s) of interest, (c) debating feasible change (comparing models to real world), (d) suggesting and improving. SSM can be iterative and it is intended for groups to engage in thinking about complex situations to reduce the likelihood of unintended consequences. SSM has successfully been used in a number of healthcare settings including developing context dependent tools to support chronic disease management [19, 20].

CCM is an approach that assists analysts and stakeholders reason about the health care system in a patient-centric manner. It builds on SSM, UML and patient journey mapping.

## Methods

The CCM was developed as part of a study exploring continuity of care for end of life patients [21]. A novel method was required to explicitly describe the healthcare system in a patient-centric manner to better understand continuity. CCM takes a systems approach to reasoning about healthcare.

A project that uses circle of care modelling will begin with an initiation step where the initial, high level goals and scope are agreed upon. The goals and scope may be refined, but it is important to have a common understanding of sense of effort and the direction being examined. CCM has its roots in Soft System Modelling and follows a similar, iterative four-step process for reasoning about a problem space. While the steps are described in sequence, these can be approached iteratively.

### Stage 1: finding out

The goal of CCM is to iteratively discover and build an understanding of the health system that is being defined (i.e. the circle of care). In the finding out stage, one scopes in or out aspects of care in more detail that are of interest and develops an appreciation for the specific health domain (s) that are considered part of the circle of care.

#### Draft personas

The CCM modeler develops an initial set of *patient personas*: patient cases that will help participants have a common viewpoint to better describe and understand the problem space. Personas are crafted to support the decisions that need to be made. Personas are initially lacking local details, but they are purposefully crafted so that aspects of the personas will help participants describe the details of challenges. Where possible, evidence (published or locally generated) can be used to inform the personas. For each persona it is helpful to highlight specific scenarios: clinical or health related vignettes linked together through time. This way challenges can evolve and multiple issues can be wrapped into a single persona.

#### Find out local details

The initial personas and their scenarios are used to structure engagement with participants. This can be completed through interviews or discussion groups. The initial personas serve as a skeleton framework that participants (patients, care givers, providers, and other stakeholders) can relate with and flesh out details from their perspectives. In sessions, they are encouraged to provide details of what might happen in each scenario. It is often helpful for the initial personas to describe the needs and high-level actions, letting the participants describe how they see that need being addressed. For example, a persona’s scenario may describe a need such as: “it is a Saturday evening on a long weekend and Mrs. Cann has run out of her pain medications. She is not sure she can wait until Tuesday.” Participants will then flesh out local details such as: “if she has a family doctor in our call group, the pharmacist can just call the doctor on call” or “we no longer have call groups, so she would end up in emergency to get that prescription, which would likely take hours”. The CCM modeler will discover that each participant may have a different view of the issue and how it can be resolved. Finding out concludes when a level of saturation occurs, that is, when the modeler develops a sense of “data adequacy” [22] with respect to two factors: 1. Role saturation – new roles of involved in the circle are no longer being discovered during interviews. 2. No new data is presenting itself in terms of communication activities or key challenging scenarios related to the topic in question. This work should be sufficient in detail needed for the scope of the project. A smaller project can more quickly limit the finding out phase to a few key informants while a larger project might need to complete this finding out in multiple locations/communities to better understand differences across a large organization. Further, as analysis occurs (e.g. early modelling), the analyst can iterately return to finding out the local details.

#### Synthesis through rich pictures (Fig. 1)

**Fig. 1:**
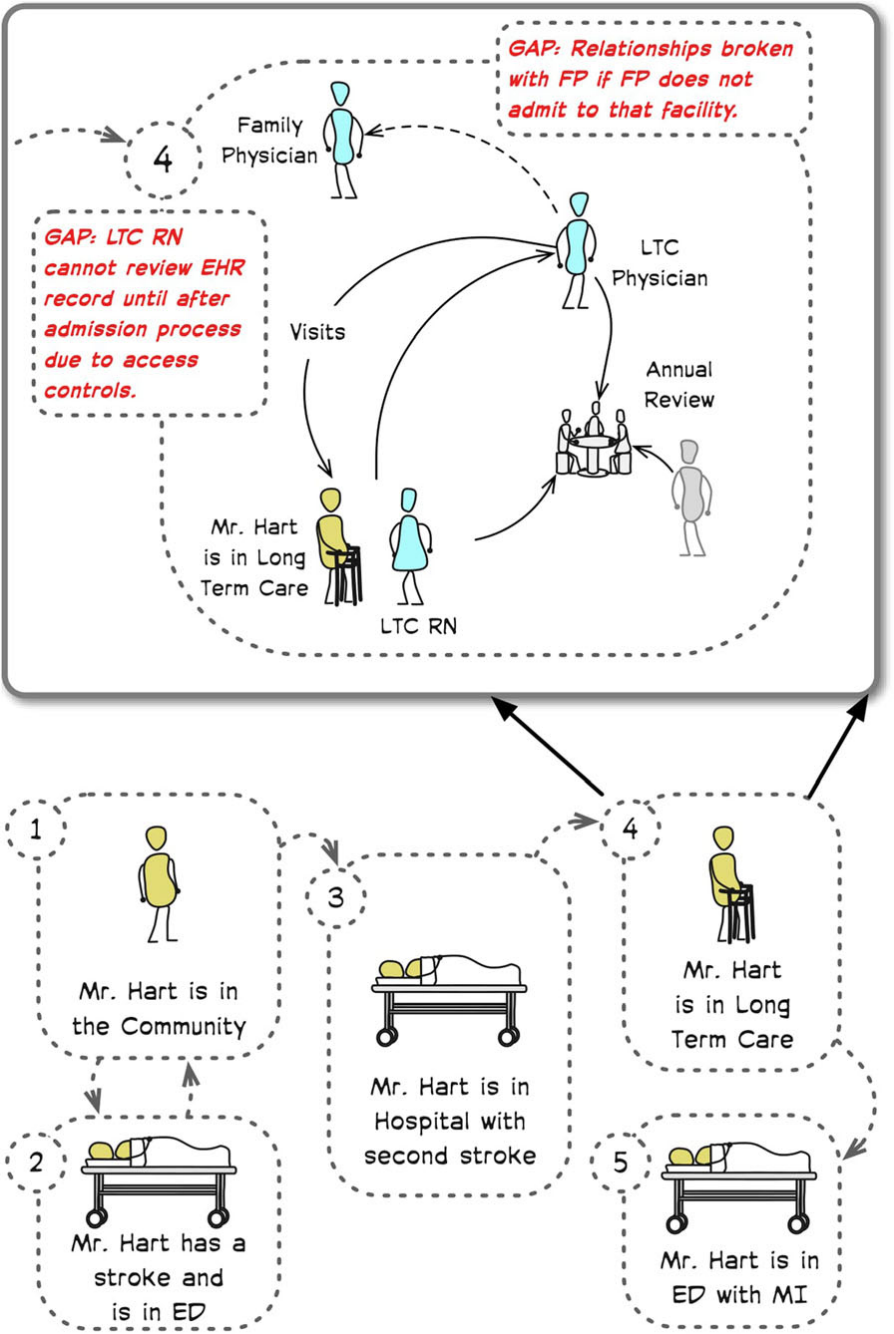
A rich picture illustrating the initial (or skeleton) framework of the persona Mr. Hart that was used in the early finding out and the more detailed illustration showing scenario 4 the fleshed out details from the analysis

As the CCM modeler reviews the data from multiple participants, they synthesize findings and develop the personas in more detail into a set of *rich pictures. Rich pictures* are analogous to patient journeys. They highlight care processes, challenges, and people involved with the patient. These rich pictures may be visual and illustrate care flows through the scenarios or textual or a combination of the two (Fig. 1). They highlight the understanding of the issues, combining insights from across the participants. The synthesis is validated with participants to ensure the rich pictures sufficiently capture the issues.

Initial target groups may be used first and then expanded to other roles as needed. Depending on the goals of the project, we have found snowball sampling [23] to be a useful method in finding out, discovering additional roles/people to engage through the finding out until saturation is reached. Snowball sampling allows for the participants to guide the research by naming roles (e.g. home care nursing) that would be involved in scenarios being described and modeled. Participants are then recruited that fill those roles. Parallel finding out processes can also be considered if comparisons are needed. For example, the same skeleton personas can be used in two communities. The resulting rich pictures may describe very different situations.

### Stage 2: conceptual modelling: three views

From the rich understanding, three views of the circle of care are modelled: a provider view, a communication view, and an information/repository view (described below). The three views were selected for two key reasons: first, they cover the main elements of a health system that are particularly relevant to health information system design and implementation. Second, multiple views helps highlight types of issues. By highlighting the people involved, their communication patterns, and the information repositories, we have a modelling approach that has a high cognitive fit [24] to some of the key problems of implementing health ICT. The patient persona is in the centre of the models and separate models are developed for each persona. Further, multiple versions of the models can be developed if comparisons are needed. These versions could be, for example, between communities/regions or between current and expected future states.

#### Provider view (Fig. 2)

The provider view highlights each of the providers in the circle, with the patient in the centre. Nodes represent providers and the edges highlight relationships between providers. The provider view shows all providers that are within a patient’s circle of care. Providers can include formal care providers (MDs, RNs, Pharmacists, etc.) and informal care givers (family, friends), depending on the purpose of the CCM. The intention of this view is to provide the analyst and stakeholders a view of the scope of people involved in care to reason about who might need to be engaged or where challenges might exist. We have found it helpful to sometimes visually cluster providers along organizational lines or along care pathway lines and to highlight provider roles linked to activities being explored (e.g. prescribing).

**Fig. 2:**
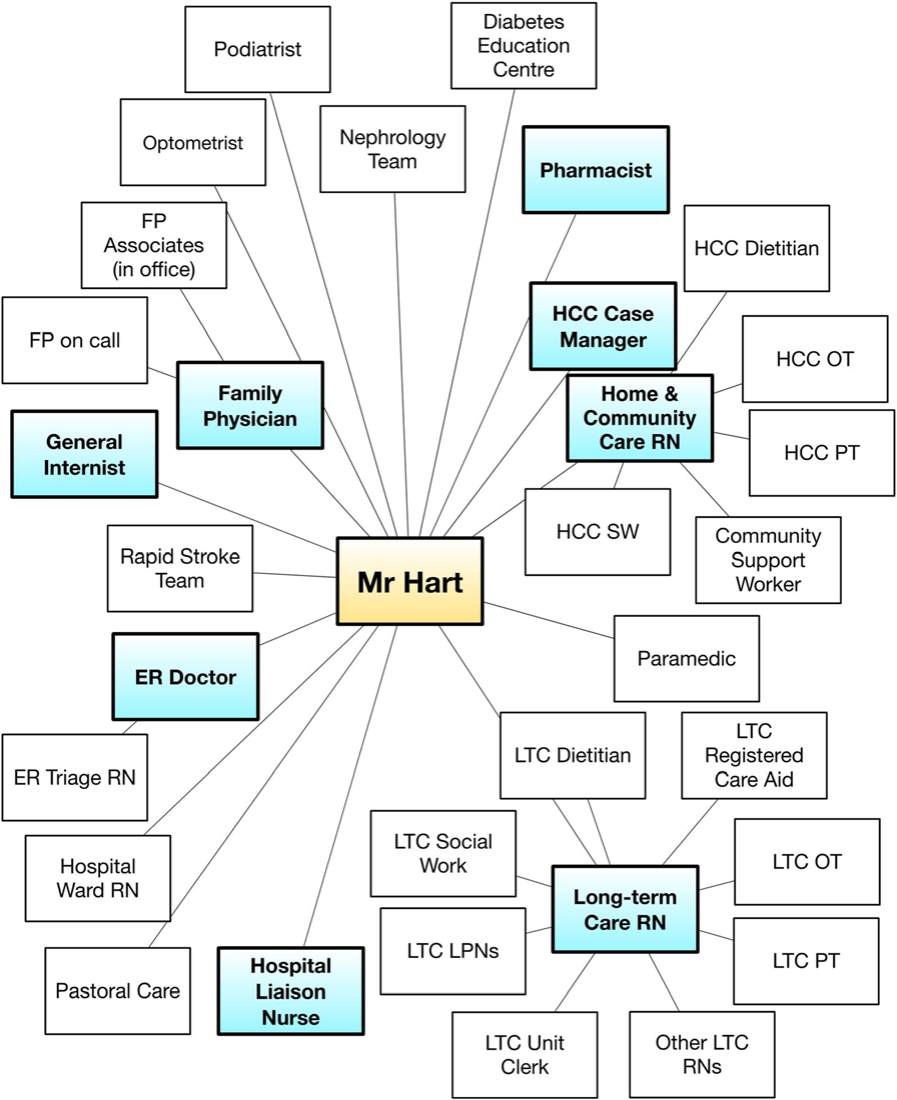
Provider view of the circle of care for Mr. Hart showing roles connected to the persona based on what was highlighted by participants. For this study, those roles highlighted in blue were most involved in inter-organizational communication

#### Communication view (Fig. 3)

The communication view highlights and describes the types of communication that occurs between members of the circle of the care. The intention of the communication view is to highlight key communication activities that are the focus on a particular issue that is being addressed. For example, communication may focus solely on aspects related to medication management or it may relate to symptom detection and assessment. The communication views will serve to highlight the problem being explored. Depending on the level of detail needed for a particular activity, clustering and theming communication activities into groups can reduce the detail and make the communication view more accessible. The communication view is derived from the UML 2.0 communication diagram. However, it has been helpful to not always include timing/order of communication on the diagram. There can be considerable variation in the order of communication between providers.

**Fig. 3:**
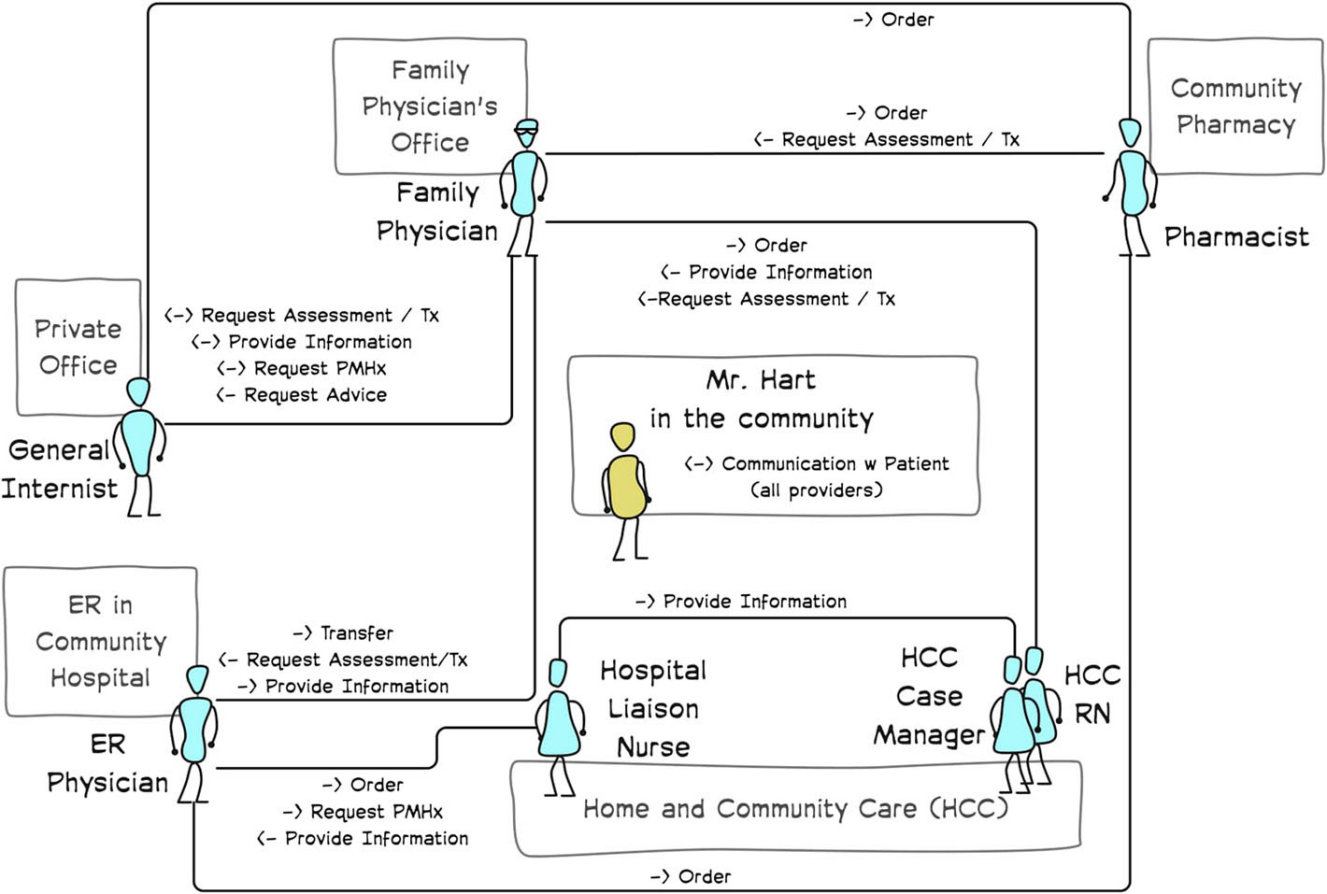
Communication view for Mr. Hart showing only the types of communication between providers and Mr. Hart while still living in the community

#### Repository view (Fig. 4)

The repository view contains all records that actively contain information on the persona. This includes paper and electronic records, local and jurisdictional, as well as those that are patient controlled and those that are informal (e.g. family health journals). The repository view is illustrated as a UML 2.0 deployment diagram with nodes being the records. Connections between nodes are used to represent information flows (e.g. when lab results are sent to a physician office or referral information is faxed to a specialist).

**Fig. 4:**
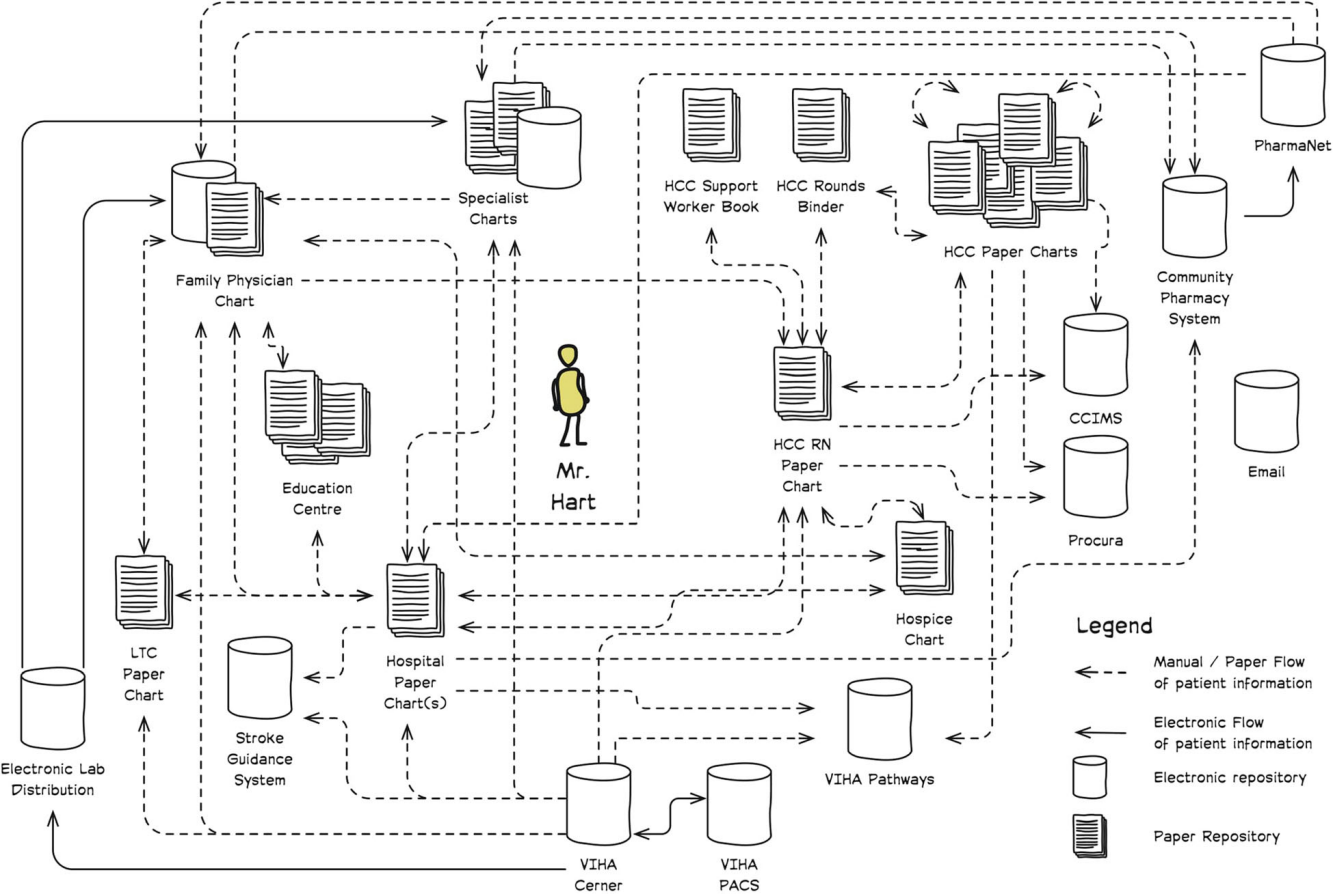
Information/Repository view of all active records for Mr. Hart. This highlights expected information flows, which could be described if needed (e.g. lab results)

### Stage 3: structured discussion

The personas (rich pictures and conceptual models) are used to provide a structure to the discussion with stakeholders. Ideally many different groups are involved in these semi-structured discussions to spur debate and enable future buy in. For example, a group could include patients, care givers, care providers, hospital administration and members of the ICT department. Although there is potential challenges with mixed groups (especially with power over relationships), the tendency to have homogenous discussion groups is not recommended. The divergent view points are important to have at the same table [25]. Facilitation of the discussion groups becomes increasingly important in mixed groups. The personas and scenarios are useful for ensuring the conversations are taken out of personal experiences and we reflect back discussion to the personas as we explore challenges and solutions. We have also used patients who have been trained to be patient representatives for health system change to participate in discussion groups. If multiple discussion groups are needed, it is recommended to structure them so that each one has a breadth of stakeholders.

The nature of the discussion can vary, depending on the state of the project. In early requirements engineering, the discussion could focus on highlighting and agreeing on the issues that need to be addressed. In later stages, various specific options can be compared, examining expected changes for options in the context of each persona. The discussion should reflect back to the personas, capturing benefits, challenges, risks that options present (or do not present) to the personas rather than to the stakeholders. In this way, the patient-centric nature of circle of care modelling is maintained.

### Stage 4: describing potential improvements

In the fourth stage, the potential improvements are described and reviewed. This can be in the form of documentation, visualization, or captured in discussion. It can be helpful to revisit the personas to describe the intended benefits/changes resulting from potential improvements. The broader the impacts are considered the greater the likelihood of uncovering unintended consequences.

While these are described as four linear stages, they can be applied iteratively or in a different order, as needed by the project. Some projects have pre-defined options that need to be considered and in those cases the options are described first (stage 4) followed by finding out and then modeling and discussion. In other projects, the scope is not well defined at the start and additional finding out is required. This can sometimes require new personas to be developed. These are all encouraged with the CCM approach to better fit the need of the particular project.

Each of the case studies described below received prior ethics approval through the University of Victoria.

## Results

Three CCM case studies are described next. For each case, we will state a goal and then describe application of CCM through its four stages.

### Case 1: improving continuity of care for end of life patients [21, 26]

#### Goal

The goal of this study was to discover feasible ways legacy health ICT could be changed to improve continuity of care for end of life patients.

#### Finding out

Two personas were developed from provincial end of life care data [27]. Each persona had four scenarios highlighting transitions in care needs. Snowball sampling was used to interview 34 participants across two communities in the same health region (including 6 ICT staff). Interviews started with GPs, who then named other roles involved in maintaining continuity. Rich pictures were made for each persona in each community that highlighted gaps in care that were described from multiple participants. An additional scenario was added through iteration and reflection on participant feedback.

#### Conceptual models

Models were made for each persona in each community. Many of the issues were different, due to differences in health resources in the larger community.

#### Structured discussion

Two focus groups were held with eight participants, one in each community. Feasible options for improvement (defined as being implementable within 12 months with ICT leadership) were discussed and validated in each group.

#### Potential improvements

Six suggested improvements to continuity were described that ranged from improved access to clinical information systems to automatically notifying providers in the circle of care to transitions in care (e.g. admissions/discharges). The latter has since been implemented for the region.

#### How CCM helped

CCM was helpful to participants in this study in that it surfaced regional issues as well as cross-organizational issues that were not previously explicit. For example, CCM helped make clear to decision makers how an individual’s health data was fragmented across various care teams and in multiple electronic and paper records across the region. Decision makers were not aware of the extent fragmentation and CCM made this clear. By showing what care providers were involved in a patient’s care, decision makers were also able to see complexities in communication as well as gaps and duplication of services.

### Case 2: communication related to medication management [28]

#### Goal

To develop a communication model that captures stakeholders involved in medication communication that can be used to reason with when considering impact of new features for a jurisdictional drug information system.

#### Finding out

This study was developed with three iterations, each of which completed the four stages of CCM: 1. Literature review on medication communication, 2. Deep analysis within a single community health centre (with an integrated pharmacy), and, 3. More broad engagement and interviews with participants in a larger community. Three personas were used to structure the discussion that had scenarios of medication renewals, specialist, hospital and walk in clinic use as well as use of multiple pharmacies over time.

#### Conceptual models

Conceptual models were developed for each persona and revised through each iteration.

#### Structured discussion

Structured discussions were repeated for each iteration: 1. within the study team, 2. with the community health centre, 3. with participants from the community.

#### Potential improvements

The structured discussion in iteration two focused on local improvements for the community health centre while discussions in iteration 3 provided suggestions more broadly to how the jurisdictional drug information system could better support medication communication with a broader range of providers (family doctors, office staff, specialists, pharmacists).

#### How CCM helped

CCM was able to highlight and describe in detail the considerable efforts required for outpatient medication coordination across the circle of care between organizations. This previously as been relatively under-described and was highlighted through CCM modeling. This has implications as new regionalized systems are being developed to better support medication management.

### Case 3: improving attachment of patients to primary care [29]

#### Goal

To decide on options to improve attachment of patients to primary care in a community. CCM was applied in a low-cost and rapid manner: CCM was used to structure four evening focus groups (2–3 h each) to help the community discuss options. The study began with five potential options: 1) A community health centre for unattached patients, 2) Enhanced home care services for chronic disease, 3) Integrated additional RN services primary care, 4) A hospital based urgent care clinic, and 5) GP office efficiency improvements/coaching to increase in office capacity. Three analysts facilitated four large discussions in the community with physicians, home care, the health region and patient representatives (30+ participants) over 4 months.

#### Finding out

There was a local health needs survey data that was collected prior to this study that was used to create five patient personas and their initial rich pictures who had varying amounts of attachment to primary care. These were validated in the first focus group with all participants and adjusted based on feedback.

#### Conceptual models

Conceptual models were created to highlight how each of five health systems changes could impact care for each of the personas between the first and second focus groups for each persona.

#### Structured discussion

For the second and third discussion groups, the personas were used to structure the discussion. Each of the five options were reviewed in the context of each persona. The relative benefits/challenges for each option were discussed for each persona. One researcher facilitated the discussion and two researchers actively documented findings and clarified comments during each session.

#### Potential improvements

In the fourth discussion group, the findings were reviewed and confirmed with participants. Poorly attached patients could benefit from a range of services. A community health centre would likely best support unattached patients.

#### How CCM helped

CCM was effective in this instance in engaging a large group of over 30 stakeholders to discuss a range of potential changes to the health care system, each proposed by a different stakeholder group (e.g., GPs, Emergency, Homecare). The personas and models provided the structure needed to take discussion away from departmental requests to reasoning about expected benefits/challenges for patients for each option. This took the discussion away from organizational needs to patient needs and structured the discussion around patient benefits (for types of patients) and describing gaps that would or would not be addressed by the proposed options. CCM encouraged the group to consider which types of patients they would need to focus on first and then consider which health system change would be most effective.

## Discussion

The need for increased patient centeredness in health care is clear [30, 31]. The CCM approach has been successfully used in several projects addressing early exploration of healthcare system change. Unlike many other modeling approaches, CCM is explicitly designed to structure discussion with stakeholders so that debate is patient-centric and systems oriented. This is achieved through using patient personas and visualizing the circle of care (i.e. the patient’s own healthcare system) through a set of models. CMM is built on concepts from SSM, UML, and SysML, and, like them, CCM includes multiple views that highlight the system in question in different ways. CCM includes views for describing process (the rich picture and communication view) as well as structure (the provider and information/repository views). However, CCM is an improvement in terms of patient-centeredness over these other methods as it explicitly contextualizes the description and modelling with the patient in the centre and uses the circle of care as the boundaries of the system being modeled.

Analysts can use other methods and intentionally keep a focus on the patient, but CCM incorporates patient centeredness by design. More general modeling approaches such as UML or Soft Systems *can* be used in a patient centric manner, they are not always used in this way. This can be true even when a project has a guiding principle of being patient centric. Patient centeredness may be lost, particularly in large and long projects with multiple stakeholders. Specialization can be helpful for practitioners. Just as SysML is a specialization of UML, so CCM can be considered a specialization of SSM. CCM’s strength, then, is that it is an approach that instantiates the high-level principle of being “patient centered” into a tangible practice. CCM keeps the patient explicitly in the centre from investigation, to analysis, visualization, discussion and through to decision making. Analysts and stakeholders in a healthcare project can use CCM to reason about changes keeping the patient central and explicit in the process. CCM surfaces tacitly held knowledge about patient care to decision makers. Thus, analysts “in the trenches” have tools to ensure the principle is practiced in the project.

**Fig. 5:**
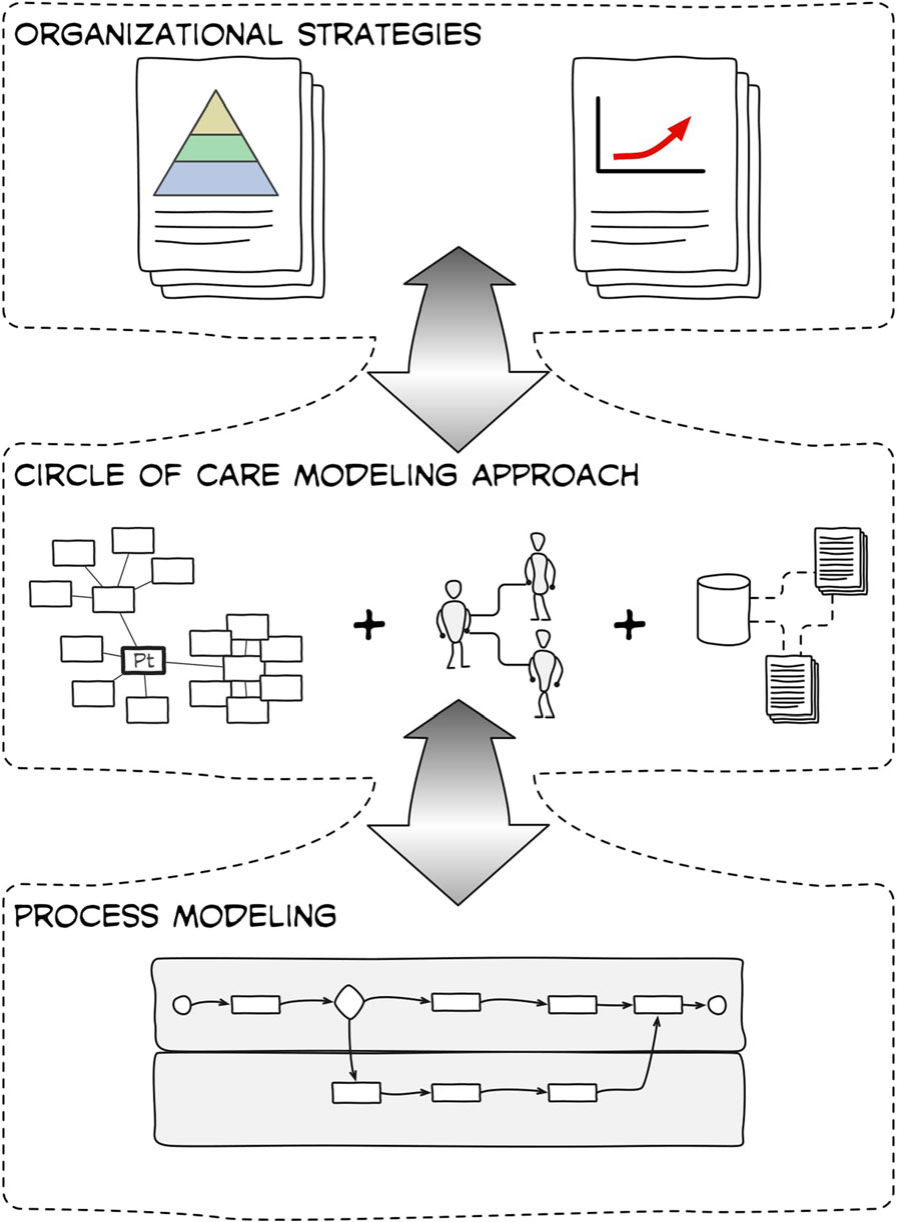
CCM is positioned as a modeling approach to help address healthcare system problems at a “meso” level. It both crosses organizations and maintains a level of detail to highlight local issues

CCM is also designed to analyze and explore cross-organizational issues. CCM is a “meso” level approach: it is more detailed than strategic plans (macro level) and broader than detailed activity modeling (micro level) (see Fig. 5). As such, it is better suited to describe complex issues and thus help analysts understand complex issues that impact care of patients that are broader than optimization of specific workflows. For example, CCM is well suited to explore issues of continuity, information access, or multi-organizational shared care as it structures the stakeholder discussion around patients in a way that the analysis makes explicit some of these challenges.

Patient journey mapping is patient centric is an approach that is similarly patient centered. Indeed, CCM’s Rich Pictures are similar. However, CCM includes more structural views (e.g. provider and information/repository views) that can help explore and highlight additional issues. Further, as the circle of care is used, CCM structures analysis and discussion more broadly than patient journey mapping. Where patient journey mapping can be focused on issues and flows to streamline processes inside an organization to make processes more lean [15], CCM encourages analysis across the circle of care. Thus, CCM is a better fit when addressing broader issues.

Being patient centric in its design and application helps participants reason more about expected patient benefits for proposed clinical changes instead of focusing on specific product features or provider, departmental or organizational benefits. That is, it can be easier for people to reason over which option might help “Mrs. Cann” or “Johnny”, even if it means that their personally preferred option (e.g., having more staff in their own department) is not the better option to help patients. Further, we have found that CCM also assists providers and other stakeholders remember the key yet informal roles of family and friends in managing care for some patients as these become part of the models that are used when discussing issues.

### CCM process adaptation through application

CCM has been applied with different methods from detailed sequential interviews to a rapid series of discussion groups. While this shows that CCM is adaptable, it does make it harder for a practitioner to adopt “the method”. Soft systems began as a prescriptive seven step process that evolved to a more flexible, four step approach [25]. In a similar way, consider that CCM is an approach with characteristics; CCM is:

1. Patient centric. The centre of discussion is focused on care or health of the patient (with their family). The scope of the circle includes formal and informal caregivers, tools, technologies, organizations, activities and other aspects relevant to the change being considered, but it starts and ends with patients.
2. Systems based. The system is the circle of care. This encourages participants to consider the proposed changes in the context of a connected system. The system existing within its environmental context and constraints.
3. Change focused. CCM is used to explore changes. The changes could be explored prospectively, as the case studies do, to explore expected change. Changes could also be examined retrospectively.
4. Visual, using personas, rich pictures and circle of care models as tools. The models are visual thinking tools that encourage participants to focus on the goals and issues highlighted. Personas do not need to be representative, models do not describe all the details. Instead, they visually and intentionally highlight the aspects that need to be explored.
5. Evidence-based, iterative, and reflective. Use evidence in its many forms, including the evidence gathered through the CCM process to inform reasoning about the issues. External evidence can be useful, but not always transferrable. Local evidence can be extremely helpful and should be developed as part of finding out.

### Future work

Continued application of the CCM is important for further refinement. A future direction could be to use real patient data to generate the models. This could be explored with specific patients being modeled as specific index cases, for example. Circle of care models could also be developed more quantitatively using populations of patients and mining data from existing records. This would open up the possibility to apply quantitative social network analysis methods within the circles of care to better quantify communication and information patterns. Additional consideration to the temporal aspects of care would be important to consider in future work. Rich pictures provide a storyline but the other models do not capture how a circle of care can change. Being able to describe and show changes to the circle of care over time (e.g., with a new diagnoses) is an area for future research. Comparative studies, exploring the same challenges with CCM and other traditional methods to show how CCM results differ from other approaches. would be important future work.

A remaining consideration is how far can one scale CCM down while still adding value to a requirements process. In some circumstances there may not be the capacity (resources or time) to work through multiple iterations with a broad range of stakeholders. Thus, ways of keeping CCM a “light” method would be valuable. Case #3 highlighted one approach where 3–4 focus groups were held with interested community members, leveraging existing information and forgoing a more detailed finding out phase with many interviews. Another approach would be to recruit a smaller group of participants that would be expected members of the circle of care and co-develop the rich pictures and models, leveraging some of the co-creation methods that are increasingly popular in design research, outside of healthcare. Future fieldwork is needed in this area.

## Conclusion

Patient centeredness is key for health care but, with the complexity of many healthcare organizations and health ICT projects, it is often lost in planning of ICT projects. Instead, the organizations or the ICT systems themselves become the centre of planning and modeling and this can lead to gaps in understanding with unintended consequences with negative impacts on patients [4, 32]. This paper presents circle of care modelling, which was designed to explicitly keep the patient central as stakeholders reason about healthcare system changes. Unlike other modeling approaches, it facilitates the exploration of inter-organizational and inter-ICT system issues that would impact patient care while keeping the patient central. Thus it is better suited than other modeling approaches to seeking improvements to complex problems such as continuity, attachment, and multi-organizational health system processes. While this paper describes the use of CCM on health ICT projects, it can be used for non-ICT related projects that require systems thinking.

## Abbreviations

CCM: Circle of care modelling
ICT: Information and communication technology
MD: Medical doctor
PHP: Patient health portal
PHR: Personal health record
RN: Registered nurse
SysML: Systems modeling language
UML: Unified modelling language
